# Thermal stability data of juglone from extracts of walnut (*Juglans regia*) green husk, and technologies used to concentrate juglone

**DOI:** 10.1016/j.dib.2019.104081

**Published:** 2019-05-30

**Authors:** Eduardo Caballero, Carmen Soto, John Jara

**Affiliations:** Centro Regional de Estudios en Alimentos Saludables (CREAS), Chile

**Keywords:** Thermal stability, Juglone, Concentration technologies, Hydroalcoholic extracts

## Abstract

The data presented in this article are focused on thermal stability data of both juglone standard (in ethanol and methanol) and a natural extract containing juglone from lyophilized walnut green husk (in ethanol and methanol). On the other hand, we also show the data of the impact of three concentration technologies over the concentration yield of juglone from the natural extract in ethanol and methanol. All data presented are related with the information included in “Polyphenolic extracts of walnut (Juglans regia) green husk containing juglone inhibit the growth of HL-60 cells and induce apoptosis” Soto-Maldonado et al., 2019, where the discussion and interpretation of results can be found.

**Table:** Specifications table

Subject area	*Process Chemistry and Technology*
More specific subject area	*Extraction of bioactive compounds from vegetable matrix.* *Agro industrial waste valorization.*
Type of data	*Figures and Table*
How data was acquired	*Juglone concentration was obtained by high-performance liquid chromatography (HPLC, Perkin Elmer 200 series) using a photo diode array (PDA) detector. Concentration of extracts was carried out by Rotavapor, Speed Vacuum, and Vaccum Oven equipments.*
Data format	*Raw Analyzed data*
Experimental factors	*The factors taken into account to generate the data of thermal stability were: extraction solvent, temperature, and time. In the case of concentration yield of juglone, the main factor was the concentration technology.*
Experimental features	*Data of thermal stability were obtained at four different temperatures (20°C, 40°C, 60°C and 70°C) during 8 hours every 1 h. Both, the juglone standard and natural extract (from walnut green husk) containing juglone were obtained in ethanol or methanol before to make the thermal stability test. The temperature to probe the concentration technologies was 40°C using ethanol or methanol and three concentration technologies (Rotavapor, Speed Vacuum, and Vaccum Oven equipments).*
Data source location	*The walnut green husk used for the study, were obtained 170 days after flowering, from a walnut plantation in the Libertador General Bernardo O'Higgins Region (33°56′00″S 71°50′00″W; Chile).*
Data accessibility	*Data are with this article.*
Related research article	*Soto-Maldonado, C., Vergara-Castro, M., Jara-Quezada, J., Caballero-Valdés, E., Müller-Pavez, A., Zúñiga-Hansen, M.E., Altamirano, C. 2019. Polyphenolic extracts of walnut (Juglans regia) green husk containing juglone inhibit the growth of HL-60 cells and induce apoptosis. Electronic Journal of Biotechnology, 39: 1–7.*

**Table:** 

**Value of the data** • The data present the information about thermal stability of both juglone extracts from walnut green husk and juglone standard at different temperatures and solvents. • Data of thermal stability of juglone standard from chemical synthesis and juglone extract from green husk walnuts (natural resource), can help to the researchers to investigate the factors that determine the differences between them. • The data of concentration yield of juglone when different concentration technologies were used are valuable to improve and develop new industrial technologies to apply for bio-refinery of bioactive compounds.

## 1 Data

**Fig. 1:**
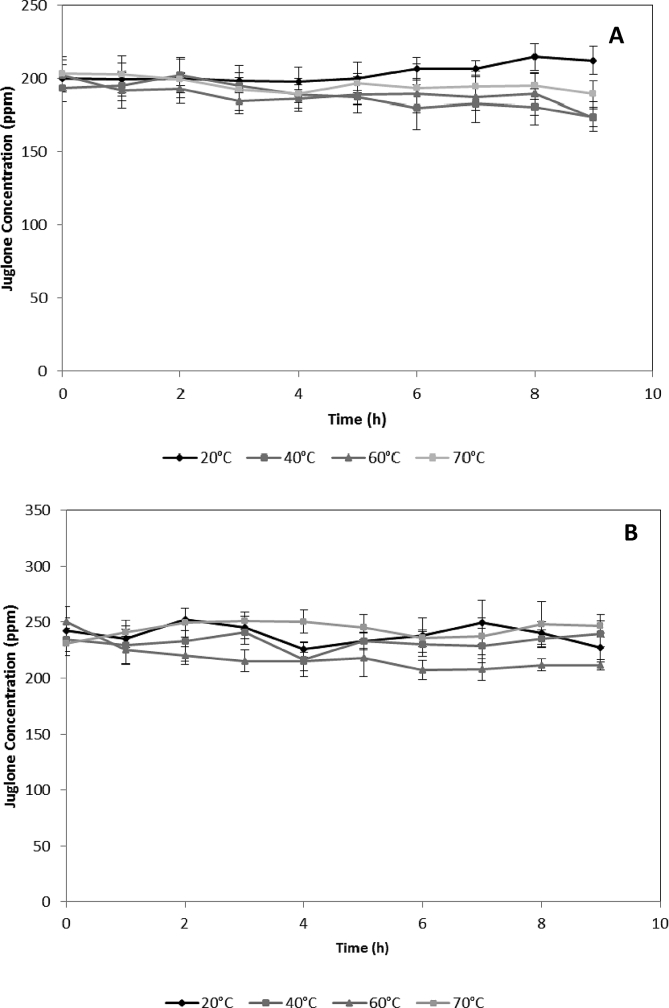
Thermal stability of juglone standard at different temperatures. **A**: juglone standard in ethanol, **B**: juglone standard in methanol.

**Fig. 2:**
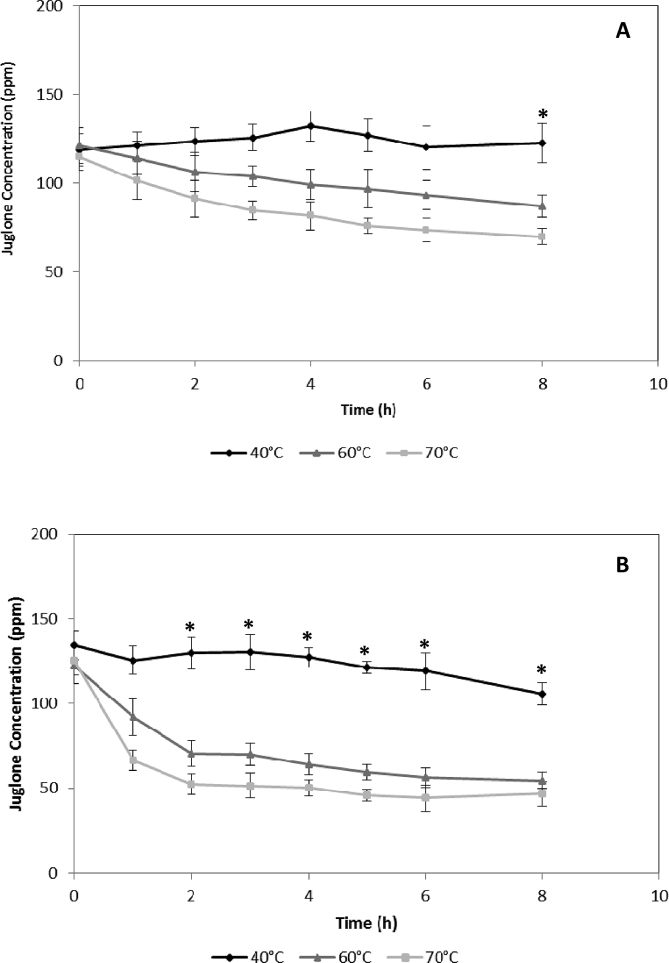
Thermal stability of juglone extracts from walnuts green husk at different temperatures. **A**: juglone extract in ethanol, **B**: juglone extract in methanol. * Statistically significant difference with a P value < 0.05.

Data described here are related with the kinetics of thermal stability of juglone standard in ethanol (in a range between 173.6 and 212.6 ppm of juglone) and methanol (in a range between 206.9 and 251.3 ppm of juglone) at 20 °C, 40 °C, 60 °C, and 70 °C (Fig. 1) and juglone extracted from walnut green husks in ethanol (in a range between 70.1 and 132.2 ppm of juglone) and methanol (in a range between 44.2 and 134.5 ppm of juglone) (Fig. 2) at 40 °C, 60 °C, and 70 °C. Data from Fig. 1, Fig. 2 are useful to estimate the shelf-life, storage conditions, or concentration conditions of the extracts and if there is any difference between the juglone extract and juglone standard at the same temperatures. Besides, the data of juglone yield using three concentration technologies: rotavapor concentrator (RV), speed vacuum (SV), and vacuum oven (VO), were evaluated for the natural extract from walnut green husk (Table 1).

**Table 1:** Data of juglone yield from three concentration technologies using juglone extract from walnut green husk in ethanol.

Concentration Technology	Juglone Concentration (ppm)	Juglone Yield (%)
SV	2.94 ± 0.141^b^	0.14
VO	23.21 ± 1.909^b^	1.16
RV	58.07 ± 4.130^a^	2.81
Control (time 0)	103.43 ± 5.402^a^	100

a Anova test determined statistically significant difference with all other concentration technologies.

b Anova test determined statistically significant difference with RV and Control but not with SV or VO respectively.

## 2 Experimental design, materials, and methods

### 2.1 Materials

The walnut green husk samples was stored frozen and then lyophilized using an Ilshin freeze-dryer (−53 °C; 50 mTorr). The lyophilized sample was processed using an IKA A 10 Basic mill to obtain a powder and then vacuum packaged until its use. Juglone standard (99% purity) was purchased from Sigma-Aldrich.

### 2.2 Thermal stability tests

The powder from walnut green husk/solvent (ethanol or methanol) ratio used to obtain the juglone extracts was 1:20 at 40 °C for 6h [2]. The supernatant was analyzed by HPLC to quantify the juglone concentration. The extracts were incubated at different temperatures (40 °C, 60 °C, and 70 °C) for a maximum period of 8h. Samples were taken every 1h and filtered using a 0.45 μm mesh to obtain walnut green husk extracts to measure the juglone concentration by HPLC [1]. The data of juglone concentration in a period of 8h was graphed at each temperature for the extracts in ethanol or methanol to obtain the kinetics of thermal stability. The same methodology was used with juglone standard at 20 °C, 40 °C, 60 °C, and 70 °C in ethanol and methanol during 9h.

### 2.3 Concentration technologies

To concentrate the juglone from green husk extracts in ethanol, three concentration technologies were used: Speed vacuum (SV), vacuum oven (VO), and rotavapor (RV). All the three alternatives were carried out at 40 °C and the juglone concentration was measured before and after concentration. The concentration process was carried out until the initial liquid extract was reduced to the 5% of the volume. The final concentrated extract was used to measure the juglone concentration. The final juglone yield of each technology was quantified in terms of the mass balance.

### 2.4 Statistical analysis

The program Addinsoft (2019). XLSTAT 2019.1.3 statistical and data analysis solution. Boston, USA, was used to carried out the ANOVA test to determine the statistically significant difference with 95% confidence.
